# Implantation of an Extravascular Implantable Defibrillator Using a Substernal Lead in a Patient with Previous Cardiac Surgery

**DOI:** 10.19102/icrm.2024.15104

**Published:** 2024-10-15

**Authors:** Alexander Breitenstein, Jean-Yves Delaite, Nicolas Dayal

**Affiliations:** 1 Electrophysiology, Department of Cardiology, University Hospital Zurich, Zurich, Switzerland; 2 Cardiology Office, Nyon, Switzerland; 3 Cardiology and Electrophysiology, Hôpital de la Tour, Meyrin, Switzerland

**Keywords:** Cardiac surgery, extravascular ICD, substernal lead, thoracotomy

## Abstract

We present the case of a 52-year-old man suffering from malignant mitral valve prolapse syndrome. He underwent a right-sided thoracotomy for mitral valve repair but required implantable cardioverter-defibrillator (ICD) implantation 4 years later. He chose the option of a substernal ICD, which was implanted successfully without any complications and good electrical parameters.

## Case presentation

A 52-year-old man was diagnosed with mitral valve prolapse in the context of Barlow’s disease, causing limitations in physical exercise tolerance and episodes of paroxysmal palpitations. His grandfather suffered from the same disease and died due to sudden cardiac death. In 2021, he consulted for an episode of syncope during cycling. Rhythm evaluation revealed frequent premature ventricular contractions (PVCs) with a burden of up to 17%, originating from the posteromedial papillary muscle and right ventricular outflow tract, with multiple short runs of ventricular tachycardia of up to five beats and a maximum rate of 204 bpm. Imaging (echocardiography and magnetic resonance imaging) confirmed the presence of mitral valve prolapse with moderate mitral regurgitation, a mitral annular disjunction of 10 mm, and significant late gadolinium enhancement in the inferior as well as inferolateral walls. The left ventricular ejection fraction was normal, as was a coronary angiogram. The diagnosis of malignant mitral valve prolapse was established according to the European Society of Cardiology (ESC) consensus document,^1^ and the patient was referred for mitral valve surgery.

For surgery, a minimally invasive approach via a right-sided thoracotomy (4–5 cm) through the fourth intercostal space was chosen. Cardiopulmonary bypass was established through femoral cannulation. The left atrium was accessed via the Waterston groove. The mitral valve was repaired using a Memo 4D annuloplasty ring (LivaNova, London, UK), with cleft closure of the P1/P2 and P2/P3 segments, as well as resection of the prolapsing A2 segment. Furthermore, the left atrial appendage was closed with a clip (AtriClip PRO 240; AtriCure, Mason, OH, USA). A post-surgical follow-up by the patient’s cardiologist confirmed a good result of the repair with mild residual regurgitation. Because of persistent PVCs, an implantable cardioverter-defibrillator (ICD) was discussed at this time with the patient, but he preferred continuous monitoring with an implantable loop recorder.

**Figure 1:**
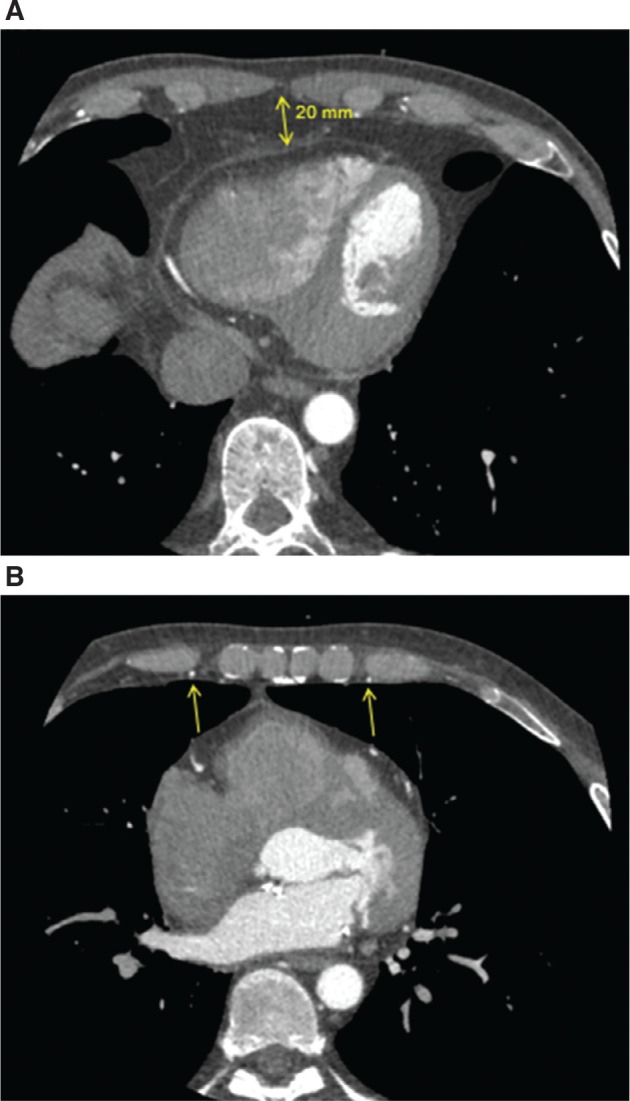
**A:** A chest computed tomography scan showing the distance between the posterior border of the sternum and the anterior cardiac structures. **B:** Note the internal mammary arteries are not in proximity to the lateral border of the sternum.

**Figure 2:**
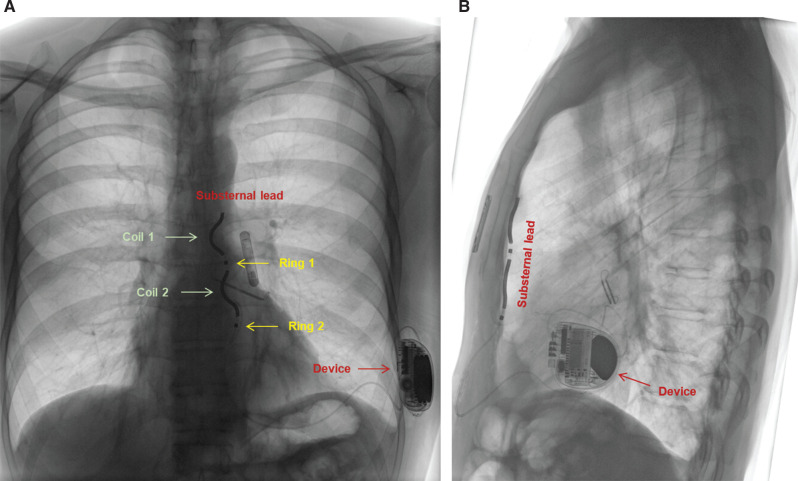
A chest X-ray taken after extravascular implantable cardioverter-defibrillator implantation showing the correct position of the lead substernally **(B)** and of the coils facing toward the patient’s right side, while the sensing electrodes (Ring 1/Ring 2) are facing toward the left **(A)**.

During the following year, three episodes of symptomatic non-sustained ventricular tachycardia were recorded, with the longest being 4 s at 188 bpm despite β-blocker therapy. After further discussion with the patient, in addition to the uptitration of anti-arrhythmic (β-blocker) medication, he accepted an ICD implantation. Owing to his relatively young age, the patient preferred to keep foreign hardware outside the vasculature, and hence the option of a subcutaneous ICD or an extravascular ICD (EV-ICD) was discussed. Despite previous cardiac surgery, he chose the substernal (extravascular) option mainly due to the benefit of the smaller device size, the potential option for antitachycardia pacing, and the avoidance of a potentially visible lead on the surface of the sternum. The implantation was performed under general anesthesia in a standard operating theater using fluoroscopy. A chest computed tomography (CT) scan taken prior to the intervention revealed a distance of >2 cm between the inferior edge of the sternum/xiphoid and the anterior cardiac structures **(Figure 1)**. Furthermore, the course of both internal mammary arteries was sufficiently distant from the lateral sternal borders **(Figure 1)**. A subxiphoid incision was performed at the usual location, and the mediastinum entered at the angle between the xiphoid and the left costal margin. Using the angulated introducer tool and biplane fluoroscopic views (anteroposterior and lateral), a 9-French (Fr) sheath was advanced substernally in the anterior mediastinum without any resistance, and the lead could subsequently be placed at the recommended position. Ventricular sensing (using the standard vector configuration Ring 1–Ring 2) was excellent, with an R-wave of 1.6 mV without a discriminable P-wave and an impedance of 494 Ω. Furthermore, defibrillation testing was successful at 30 J in the standard vector configuration. A chest X-ray performed the day after confirmed stable lead and device positioning, and the patient was discharged in good condition **(Figure 2)**. The 1-month follow-up revealed satisfactory sensing performance of the device and no complications.

## Discussion

The EV-ICD is a relatively novel treatment option for the prevention of arrhythmia-associated sudden cardiac death, with the high-voltage lead being implanted substernally in the anterior mediastinum. The Extravascular ICD Pivotal Study demonstrated a high efficacy of successful defibrillation testing as well as safety when considering peri- and postinterventional complications—specifically, no cases of mediastinitis were observed, and there was no injury to the cardiac structures.^2^ The participants of the aforementioned pivotal study underwent chest CT imaging prior to the implantation procedure to delineate the anatomy of the sternum, the extracardiac space behind the sternum, the position of the internal mammary arteries, and the edges of the lungs. Previous experimental studies have facilitated optimization of the implantation procedure tools with the current use of an angulated delivery tool to advance the 9-Fr sheath strictly underneath the sternum.^3,4^ Using corresponding fluoroscopic views in anteroposterior (0°) and lateral (90°) angulations, laceration of the pericardium and/or cardiac structures can be avoided. However, this is more difficult in patients with pectus excavatum, and this patient population therefore does not currently qualify for the implantation of a substernal ICD.

Previous sternotomy is another situation representing a strict contraindication to EV-ICD implantation.^2,4^ During open heart surgery, the pericardium is generally opened in the way of an inverted “T” from the portion near the aortic arch to the diaphragm. After surgery, the cardiac surgeon can decide to close the pericardium or leave it open. Unfortunately, there is a lack of consensus among surgeons on whether to complete closure and the best method of closure.^5^ Historically, pericardial closure at the end of the procedure has been associated with a greater risk of post-surgical tamponade and impaired hemodynamics after surgery, but this was not observed in more recent studies.^6^ On the contrary, postoperative retrosternal adhesions between the right ventricle and the posterior face of the sternum are minimized if the pericardium is closed.^7,8^ Furthermore, post-surgical pericardial closure maintains the retrosternal distance between the pericardium and the posterior sternum,^9^ thus reducing the risk of myocardial injury and therefore morbidity and mortality during repeat sternotomy.^6,10^ By analogy, even though the tunneling of the delivery tool of the substernal lead in the EV-ICD intervention does not represent a true repeat sternotomy, it carries the risk of injury to cardiac structures due to the proximity of these structures to the posterior surface of the sternum; hence, previous sternotomy represents a contraindication. Finally, the wires/cerclages used to close the sternum may represent an obstacle to easily advance the angulated delivery tool along the posterior surface of the sternum.

Performing minimally invasive heart surgery via a right-sided thoracotomy (either by a submammary, right axillary, or right posterolateral approach) offers an alternative route for many surgical interventions, including mitral valve repair.^11^ During a right-sided thoracotomy, the anterior pericardium is not injured, as access to the cardiac structures is usually via a more posterolateral right-sided pericardial incision. With such an approach, the retrosternal space is maintained intact, so substernal tunneling is theoretically possible with no adhesions or risk resulting from the previous surgical intervention.

Independently of a patient’s cardiac history, a CT scan prior to the implantation of a substernal ICD, though not mandatory, is strongly recommended. It helps to identify the anatomical location of both internal mammary arteries, which usually lie parallel to the sternal border and could be injured if the lead is placed lateral to the sternal edge. Furthermore, the size of the retrosternal space can be identified, which is of paramount importance to the safety of the intervention.^2,4^ At the more cranial end of the sternum, the visualization of the edge of both lungs is important to identify a potential risk for contact of the delivery tool with the pleura. A careful preoperative examination of the CT of our patient revealed no visible obstacles/contraindications to the implantation of the substernal lead despite the previous cardiac surgery and allowed for a straightforward implantation with no complications.

To the best of our knowledge, this is the first published case of an EV-ICD implantation in a patient with previous cardiac surgery. This case highlights why cardiac surgery from a non-sternotomy approach does not represent a strict contraindication to EV-ICD implantation.
